# The Lethal and Sublethal Effects of Lambda-Cyhalothrin and Emamectin Benzoate on the Soybean Pest *Riptortus pedestris* (Fabricius)

**DOI:** 10.3390/toxics11120971

**Published:** 2023-11-30

**Authors:** Jianglong Guo, Jingjie An, Hong Chang, Yaofa Li, Zhihong Dang, Chi Wu, Zhanlin Gao

**Affiliations:** 1 Key Laboratory of Integrated Pest Management on Crops in Northern Region of North China, Ministry of Agriculture, Plant Protection Institute, Hebei Academy of Agricultural and Forestry Sciences, Baoding 071000, China; jianglongguo@haafs.org (J.G.); anjingjie147@163.com (J.A.); liyaofa@126.com (Y.L.); dangzhihong310@163.com (Z.D.); 2 Institute of Plant Protection, Guangdong Academy of Agricultural Sciences, Guangzhou 510640, China; changyan305@163.com; 3 State Key Laboratory for Biology of Plant Diseases and Insect Pests, Institute of Plant Protection, Chinese Academy of Agricultural Sciences, Beijing 100193, China; wuchi@caas.cn

**Keywords:** bean bug, bioassays, pyrethroid insecticide, avermectin family insecticide, two-sex life table, sublethal effects, vitellogenin gene

## Abstract

*Riptortus pedestris* (Fabricius, 1775) (Hemiptera: Alydidae) is a major soybean pest in East Asia that can cause soybean staygreen syndrome. To date, no insecticides have been registered for the control of *R. pedestris* in China, and these insects are primarily controlled in the field through the application of broad-spectrum insecticides including lambda-cyhalothrin (LCT) and emamectin benzoate (EMB). Here, the lethal and sublethal effects of LCT and EMB on *R. pedestris* were comprehensively evaluated. LCT and EMB were both found to exhibit high levels of toxicity and concentration-dependent repellent effects for *R. pedestris*. The exposure of third instar nymphs from the F_0_ generation to LC_30_ concentrations of LCT and EMB resulted in a significant increase in the duration of nymph development and adult pre-oviposition period (APOP), together with reductions in fifth instar nymph and adult body weight, longevity, oviposition days, fecundity, vitellarium length, lateral oviduct diameter, and vitellogenin (*Vg*) gene expression as compared to control treatment. Strikingly, these suppressive effects were transmitted to the F_1_ generation, which similarly experienced the prolongation of preadult development and the preoviposition period (TPOP). Relative to control-treated populations, the F_1_ generation for these insecticide-treated groups also exhibited significant decreases in population parameter values. Overall, these data offer new insight into the impact that LCT and EMB treatment can have on *R. pedestris*, providing a valuable foundation for the application of these pesticides in the context of integrated pest management strategies aimed at soybean crop preservation.

## 1. Introduction

The rising need for a larger global food supply has accelerated the agricultural intensification process, leading to the widespread application of insecticides [1]. Indeed, insecticide use has emerged as a primary approach to pest control even though alternative strategies are available in many cases [2], with some areas using insecticides as the only approach to pest management. In the Thai horticultural system, for example, over half of all farms are entirely dependent on synthetic insecticides [3]. The excessive application of insecticides poses a significant threat to environmental safety and public health, in addition to contributing to the development of insecticide-resistant insects [4,5]. It is thus vital that insecticides be applied in a rational manner in order to mitigate these risks, with many recent studies having focused on the comprehensive evaluation of different insecticides used in the context of pest management [6,7].

The most common approaches to assessing the impact of a given insecticide on an insect species rely on the calculation of the median lethal dose (LD_50_) or lethal concentration (LC_50_) [8]. This approach can enable the effective comparison of the bioactivity levels of various insecticides and provides a metric for use when assessing insect resistance [9]. In agricultural ecosystems, however, insects are commonly exposed to sublethal insecticide concentrations as a consequence of the degradation and/or misapplication of these agents [9]. Sublethal exposure events have the potential to influence the biology, behaviors, and/or physiology of exposed insects, thus impacting overall population growth [5,6,10,11]. Intensive research efforts have explored the sublethal effects of insecticides including neonicotinoids, organophates, and pyrethroids [9]. These analyses have revealed that sublethal insecticide exposure can adversely impact the body weight, longevity, and fecundity of target species including *Cydia pomonella* (L., 1758) (Lepidoptera: Tortricidae) [7], *Aphis gossypii* Glover, 1877 (Hemiptera: Aphididae) [11], and *Plutella xylostella* (L., 1758) (Lepidoptera: Plutellidae) [12]. In other species, however, hormetic effects wherein certain insecticides exhibit stimulatory effects at sublethal doses have been reported. For example, significant increases in *Aphis craccivora* Koch, 1854, (Hemiptera: Aphididae) longevity and fecundity were reported following exposure to LC_10_ and LC_25_ concentrations of flupyradifurone [13]. These effects also have the potential to be transgenerationally transmitted to offspring, resulting in long-lived and potentially permanent impacts on population dynamics [7]. The rational application of insecticides in the context of an integrated pest management (IPM) strategy is thus strongly dependent on comprehensive analyses of these sublethal effects.

The bean bug, *Riptortus pedestris* (Fabricius, 1775) (Hemiptera: Alydidae), is a prominent agricultural pest species that is widely distributed throughout China, Japan, Korea, and other Asian countries [14]. These pests feed on over 30 species of plants from 13 families and exhibit a preference for leguminous plants, particularly soybeans [15]. Both nymphs and adults can cause damage to soybean pods as a result of their piercing and sucking behaviors, resulting in shriveled or empty pods and pronounced reductions in crop quality and yield [15]. *R. pedestris*-related crop damage is increasingly common in the Huang-Huai-Huai region, and these pests have been confirmed to cause soybean staygreen syndrome, which is characterized by deferred leaf and stem senescence, deformed pods, and seed abortion [16]. At present, however, no insecticides have been registered for the control of *R. pedestris* in China, and these pests are primarily controlled in the field through the application of broad-spectrum insecticides, particularly pyrethroids and avermectins [17].

Lambda-cyhalothrin (LCT) is a highly effective broad-spectrum pyrethroid insecticide that has been employed to control a range of pest species including *Apolygus lucorum* (Meyer-Dür, 1843) (Hemiptera: Miridae) [18] and *Mythimna separata* (Walker, 1865) (Lepidoptera: Noctuidae) [19]. LCT functions by targeting sodium channels to induce membrane depolarization and inhibit action potentials, resulting in spasms, paralysis, and death [20]. The avermectin family insecticide emamectin benzoate (EMB) is also a common agent used to control pests in a range of crop ecosystems [21]. EMB serves as an agonist that can stimulate gamma-aminobutyric acid (GABA)- and glutamate (Glucls)-gated chloride channels, resulting in neuromuscular paralysis and death [22]. In addition to their direct lethal effects, both LCT and EMB have been reported to cause sublethal and transgenerational effects in species including *M. separata* [19], *Mamestra brassicae* (L., 1758) (Lepidoptera: Noctuidae) [23], and *Spodoptera littoralis* (Boisduval, 1833) (Lepidoptera: Noctuidae) [24]. No studies to date, however, have explored the sublethal effects of LCT and EMB on *R. pedestris*. The irrational application of insecticides may cause the resistance development and/or re-emergence of *R. pedestris*, thus failing to control this pest.

In order to explore the lethal and sublethal effects of LCT and EMB on *R. pedestris* and more rationally guide insecticide application in the field, after initial analyses of the toxicity and repellent effects of these two insecticides, their sublethal effects on developmental duration, body weight, morphological parameters, reproductive parameters, and ovarian development were assessed in exposed *R. pedestris.* The transgenerational effects of these two insecticides were further explored in these pest species. In addition, vitellogenin (Vg), a yolk protein precursor, is mainly synthesized in the fat body, secreted into the hemolymph, then taken up into developing oocytes, and plays a key role in regulating insect reproduction [25]. The expression level of *Vg* is an important parameter for assessing the sublethal effect of insecticides on reproduction [25]. For instance, the egg-laying and *Vg* expression level of *Conopomorpha sinensis* Bradley, 1986, (Lepidoptera: Gracillariidae) was significantly suppressed after LC_30_ EMB exposure; LC_25_ triazophos significantly promoted *Vg* expression level of *Sogatella furcifera* (Horváth, 1899) (Hemiptera: Delphacidae) [26]. Therefore, the expression level of *Vg* is also assessed in F_0_ and F_1_ generations of *R. pedestris*. Together, these results have the potential to provide a foundation for the more effective IPM-based management of *R. pedestris* to better improve crop integrity.

## 2. Materials and Methods

### 2.1. Insects and Chemicals

*R. pedestris* specimens were collected from soybean fields in Shijiazhuang, Hebei province, China (38°19′ N; 114°7′ E), and used to establish a laboratory culture. Nymphs and adults were reared in nylon cages (100 mesh, length × width × height = 39 × 39 × 31 cm) under controlled conditions (26 ± 1 °C, 60 ± 5% relative humidity, 16 h/8 h (L/D) photoperiod) and fed insecticide-free soybean seedlings and dried seeds. LCT (97% pure) was obtained from Beijing Green Agricultural Science and Technology Co., Ltd. (Beijing, China). EMB (83.5% pure) was from Hebei Weiyuan Biochemical Co., Ltd. (Shijiazhuang, China).

### 2.2. Bioassays

LCT and EMB toxicity levels were evaluated using a seedling-dip method akin to the previously reported leaf-dip method [5]. Briefly, insecticide stocks were prepared in acetone and diluted with water containing 0.1% (*v*/*v*) Tween-80 to six different concentrations (LCT: 20, 10, 5, 2.5, 1.25, 0.625 mg/L; EMB: 50, 25, 12.5, 6.25, 3.125, 1.5625 mg/L). Soybean seedlings with two cotyledons were dipped into these solutions for 20 s, allowed to air-dry at room temperature, and transferred into plastic containers. Control seedlings were instead dipped in dH_2_O containing 0.1% (*v*/*v*) Tween-80. The roots of these seedlings were wrapped in dH_2_O-soaked absorbent cotton, and nymphs were transferred to the plastic container, which was covered with gauze to prevent their escape. Four replicate treatments were established for each concentration, with 20 3rd instar nymphs being used for each replicate. Mortality was evaluated at 72 h post-treatment. Nymphs were considered deceased if they did not exhibit any physical response when probed with a fine paintbrush.

### 2.3. Dual-Choice Behavior Assays

To evaluate *R. pedestris* preferences for insecticide-treated soybean seedlings relative to untreated seedlings, a dual-choice behavior assay was conducted using a Y-tube olfactometer consisting of a 25 cm long central tube and two 20 cm lateral arms at a 60° angle. These two branch tubes were connected to two separate flasks as odor sources. One of these flasks contained a soybean seedling treated with the selected dose of LCT or EMB (LC_90_, LC_70_, LC_50_, LC_30_ treatment groups), with the other containing a control seedling treated with dH_2_O containing 0.1% (*v*/*v*) Tween-80 (control group). A 3rd instar nymph that had been deprived of food for 24 h was released at the base of the central arm in this Y-shaped tube and monitored for 5 min. All nymphs that traveled across 2/3 of the lateral arm and remained there for at least 5 s were considered to have made a ‘choice’. Any nymphs that did not make a choice during this period were removed and recorded as having made ‘no choice’. Every five tests, the odor sources for the two Y-tube arms were reversed to control for positional bias, and a clean Y-tube was used after every 10 tests. Individual nymphs were tested a single time, and 100 total 3rd instar nymphs were tested using this approach.

### 2.4. Analyses of the Sublethal Effects of Insecticides on the F_0_ Generation

The LC_30_ concentrations of LCT and EMB were used when testing the sublethal effects of these insecticides on *R. pedestris* using the same experimental approach as above. Briefly, 3rd instar nymphs were exposed to LC_30_ concentrations of LCT or EMB as the treatment group and dH_2_O containing 0.1% (*v*/*v*) Tween-80 as a control group. After 72 h, surviving nymphs were fed insecticide-free soybean seedlings with roots wrapped in soaked cotton and dried seeds, which were changed every 3 days. Four replicates (*n* = 50 nymphs/replicate) were established for each group. Insect development was assessed on a daily basis from 3rd instar to adult emergence. The body weight of 5th instar nymphs and adults was measured using an electronic balance (0.0001 g accuracy, OHAUS Instruments Ltd., Changzhou, China), as were the body length, thorax width, and abdomen width of adults using a VHX-1000 super-high magnification lens zoom 3D microscope (KEYENCE, Osaka, Japan). For body weight and morphology parameters, three replicates (*n* = 20 individuals/replicate) were used for each group.

Female and male adults that emerged on the same day were randomly paired and transferred into a plastic container in which they were provided with soybean seedlings and dried seeds. Twenty pairs were established as a single replicate, with three replicates per group. The longevity and fecundity of each individual were recorded daily until all adults had died.

The sublethal effects of LC_30_ concentrations of LCT and EMB on ovarian development were evaluated by dissecting the abdominal cavities of adult females from the treatment and control groups. Briefly, at 1, 3, 5, 7, and 9 days post-emergence of adults, pins were used to fix adults in a dissecting Petri dish, and the abdominal cavity was then dissected. Tweezers were used to gently remove ovarian samples, which were washed thrice with phosphate buffered saline (PBS, 137 mmol/L NaCl, 2.7 mmol/L KCl, 8mmol/L Na_2_HPO4·12H_2_O, 1.8 mmol/L KH_2_PO4, 10 mmol/L, at pH 7.4) and imaged with a digital microscope (VHX-1000, Keyence, Chennai, India). The vitellarium length and lateral oviduct diameter were measured for each insect. Three replicates (*n* = 20 individuals/replicate) were used for each age of each group.

### 2.5. Analyses of Sublethal Effects on the Traits of the F_1_ Generation

Potential carryover effects of sublethal LCT and EMB concentrations on the F_1_ generation were assessed by randomly collecting 100 eggs laid by adults from each group, including LCT treatment, EMB treatment, and control groups. These eggs were then reared on insecticide-free soybean seedlings and dried seeds, with each egg serving as a single replicate. Life table parameters for nymphs and adults, as well as developmental periods, pre-oviposition, oviposition, and fecundity-related data were recorded on a daily basis until the death of the last surviving adult. Measurements were also taken of the body weight of 5th instar nymphs and adults; body length, thorax width, and abdomen width of adults; vitellarium length; and lateral oviduct diameter. For body weight, morphology, and ovarian development parameters, three replicates (*n* = 20 individuals/replicate) were used for each group.

### 2.6. Life Table Data Analyses

Population age-specific survival rate (*l_x_*), age-specific fecundity (*m_x_*), net reproductive rate (*R*_0_), intrinsic rate of increase (*r*), finite rate of increase (*λ*), and mean generation time (*T*) values were calculated with the following equations [27,28]:

(1) $$l_{x}=\sum_{j=1}^{m}S_{xj}$$

(2) $$m_{x}=\frac{\sum_{j=1}^{m}S_{xj}f_{xj}}{\sum_{j=1}^{m}S_{xj}}$$

(3) $$R_{0}=\sum_{x=0}^{\infty }l_{x}m_{x}$$

(4) $$\sum_{x=0}^{\infty }l_{x}m_{x}e^{-r\left(x+1\right)}=1$$

(5) $$\lambda =e^{r}$$

(6) $$T=\frac{\ln R_{0}}{r}$$

where *x* is the interval in days (d), *x* = 0, 1, 2, 3, …, *m* is the number of stages, *m* = 1, 2, 3, *S_xj_* is the survival rate of *R. pedestris* from egg development to age *x* and developmental stage *j*, and *f_xj_* is the mean egg production of *R. pedestris* at age *x* and stage *j*.

The age-stage life expectancy (*e_xj_*), defined as the time an individual of age *x* and stage *j* is expected to live, was also computed as follows [29]:

(7) $$e_{xj}=\sum_{i=x}^{\infty }\sum_{y=j}^{m}S_{iy}^{\prime }$$

The age-stage reproductive value (*v_xj_*), which represents the contributions of individuals of age *x* and stage *j* to the future population, was computed as follows [30]:

(8) $$v_{xj}=\frac{e^{r\left(x+1\right)}}{S_{xj}}\sum_{i=x}^{\infty }e^{-r\left(x+1\right)}\sum_{y=j}^{m}S_{iy}^{\prime }f_{iy}$$

where $S_{iy}^{\prime }$ is the probability of an individual of age *x* and stage *j* surviving to age *i* and stage *y*. $S_{iy}^{\prime }$ is calculated based on the assumption that $S_{iy}^{\prime }$ = 1.

Life history data for the F_1_ generation were analyzed using the TWOSEX-MSChart program [31] based on the age-stage, two-sex life table theory [28,32]. Standard error values for all parameters were estimated through the use of 100,000 bootstrap replicates. Differences among the LCT, EMB, and control groups were determined with paired bootstrap tests at a 5% significance level.

### 2.7. qRT-PCR

To assess the effects of LCT and EMB on *Vg* expression in *R. pedestris*, a qRT-PCR analysis was conducted following exposure to the LC_30_ concentrations of these insecticides (treatment groups) and dH_2_O containing 0.1% (*v*/*v*) Tween-80 (control group). Adults were collected on days 1, 3, 5, 7, and 9 after emergence for these analyses, which were performed as reported previously [33]. Briefly, total *R. pedestris* RNA was extracted with a TransZol Up Plus RNA Kit (TransGen Biotech, Beijing, China), and cDNA was prepared with TransScript One-Step gDNA Removal and cDNA Synthesis SuperMix reagents (TransGen, Beijing, China) based on provided directions. This cDNA then served as a template for qPCR using primers specific for *Vg* and elongation factor 1 alpha (*EF1α*) as reported by Lee et al. [33]. Gene expression levels were normalized to *EF1α* levels and relative expression was assessed via the comparative C_T_ (ΔΔCt) method. Three replicates (*n* = 20 individuals/replicate) were used for each age of each group.

The above methodology flow is shown in Figure S1.

### 2.8. Statistical Analysis

The log-probit model was used to establish the LC_30_, LC_50_, LC_70_, and LC_90_ values for LCT and EMB when used to treat *R. pedestris* 3rd instar nymphs. Chi-square tests were used to analyze dual-choice behavior assay results, with a presumed 50:50 distribution. Data collected for LCT treatment, EMB treatment, and control groups from the F_0_ generation, including developmental duration, the weight, longevity, body length, thorax width, and abdomen width of adults, adult pre-oviposition period (APOP), oviposition days, fecundity, vitellarium length, lateral oviduct diameter, and *Vg* expression levels were all analyzed via one-way ANOVAs with Tukey’s honestly significant difference (HSD) test, and significance was set at *p* < 0.05. To check the assumptions of normality and homogeneity of parametric analysis, Kolmogorov–Smirnov and Levene’s tests were used, respectively. SPSS 20.0 (IBM, Armonk, NY, USA) was used to conduct all statistical analyses.

## 3. Results

### 3.1. LCT and EMB Have Toxic Effects on R. pedestris Nymphs

In initial experiments, the respective LC_50_ values of LCT and EMB when used to expose *R. pedestris* 3rd instar nymphs were calculated to be 2.680 mg/L and 7.681 mg/L (Table 1), respectively. Subsequent experiments were performed using LCT and EMB at LC_30_, LC_50_, LC_70_, and LC_90_ concentrations, as appropriate.

### 3.2. LCT and EMB Exhibit Repellant Activity

**Figure 1:**
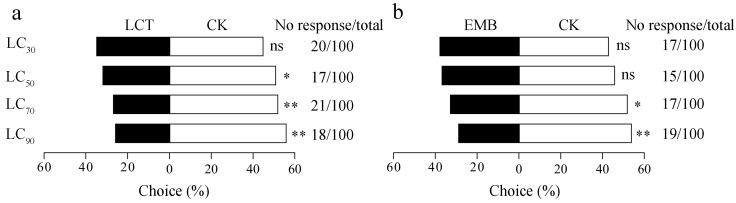
Behavioral responses of *R. pedestris* to insecticide. The repellent effects of LCT (**a**) and EMB (**b**) when used for third instar nymphs. * *p* < 0.05, ** *p* < 0.01 vs. control (CK) group; chi-square test.

When exposing soybean seedlings to progressively higher concentrations of LCT, an increase in the preference of nymphs for control group was observed such that the difference was significant for the LC_50_ vs control (*χ*^2^ = 4.35, *df* = 1, *p* = 0.04), LC_70_ vs control (*χ*^2^ = 7.91, *df* = 1, *p* = 0.005), and LC_90_ vs control comparisons (*χ*^2^ = 10.98, *df* = 1, *p* = 0.001) (Figure 1a). Similar trends were also evident for EMB, with third instar nymphs exhibiting a greater preference for control soybean seedlings as compared to those exposed to LC_70_ (*χ*^2^ = 4.25, *df* = 1, *p* = 0.04) and LC_90_ (*χ*^2^ = 7.53, *df* = 1, *p* = 0.006) concentrations of EMB (Figure 1b).

### 3.3. Sublethal Doses of LCT and EMB Impact the Developmental Duration and Longevity of R. pedestris in the F_0_ and F_1_ Generations

When third instar nymphs from the F_0_ generation were exposed to LC_30_ concentrations of LCT and EMB, the developmental durations for third (*F*_2, 497_ = 125.09, *p* < 0.001), fourth (*F*_2, 479_ = 133.14, *p* < 0.001), and fifth (*F*_2, 479_ = 47.74, *p* < 0.001) instar nymphs were significantly prolonged relative to control group. These treatments were also associated with a significant reduction in the longevity of both males (*F*_2, 177_ = 61.58, *p* < 0.001) and females (*F*_2, 177_ = 81.42, *p* < 0.001) compared to control (Table 2). LCT and EMB exposure was also associated with significantly longer development for the egg (*p* < 0.001), second instar (*p* < 0.001), fifth instar (*p* < 0.001), and preadult (egg—fifth instar) stages (*p* < 0.001) in the F_1_ generation relative to the control group, although no significant differences were noted for first (LCT, *p* = 0.60; EMB, *p* = 0.99), third (LCT, *p* = 0.91; EMB, *p* = 0.49), or fourth (LCT, *p* = 0.62; EMB, *p* = 0.69) instar nymphs when comparing developmental durations among these groups. There were also no significant differences in male (LCT, *p* = 0.23; EMB, *p* = 0.38), female (LCT, *p* = 0.21; EMB, *p* = 0.35), or overall longevity (LCT, *p* = 0.69; EMB, *p* = 0.96) when comparing the F_1_ generation LCT, EMB, and control groups (Table 3).

### 3.4. Sublethal Effects of LCT and EMB on R. pedestris Reproductive Parameters, Ovarian Development, and Vitellogenin Expression in the F_0_ and F_1_ Generations

Significant differences were observed in the APOP of the F_0_ generation among the insecticide-treated and control groups (*F*_2, 177_ = 49.23, *p* < 0.001), with the highest value in the EMB treatment group (8.67 ± 0.19 days) and the lowest value in the control group (6.37 ± 0.16 days). Significant reductions in adult oviposition days in the F_0_ generation were observed relative to control following the exposure of third instar nymphs to LCT and EMB (*F*_2, 177_ = 92.69, *p* < 0.001), with a corresponding drop in fecundity in these insecticide-treated groups (*F*_2, 177_ = 62.69, *p* < 0.001) (Table 2). While the APOP of the LCT (*p* = 0.13) and EMB (*p* = 0.31) treatment groups in the F_1_ generation was not significantly increased, significant prolongation of the total preoviposition period (TPOP) was observed relative to control (LCT, *p* < 0.001; EMB, *p* < 0.001). Significant decreases in the oviposition days of the LCT (*p* = 0.005) and EMB (*p* = 0.031) treatment groups was also observed in this generation relative to control group. Significant decreases in the fecundity of the insecticide treatment groups in this generation were also observed (LCT, *p* < 0.001; EMB, *p* < 0.001) (Table 3).

**Figure 2:**
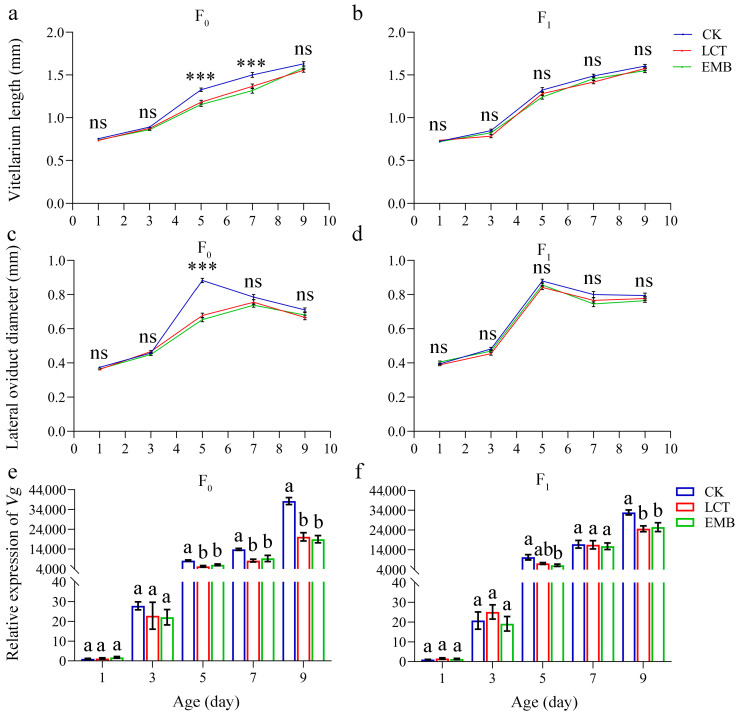
The sublethal effects of LCT and EMB on *R. pedestris* reproduction. Vitellarium lengths in the F_0_ (**a**) and F_1_ (**b**) generations. Lateral oviduct diameters in the F_0_ (**c**) and F_1_ (**d**) generations. Vitellogenin gene expression in the F_0_ (**e**) and F_1_ (**f**) generation. Three replicates (*n* = 20 individuals/replicate) were used for each age of each group. *** (*p* < 0.01) indicates a highly significant difference, ns (*p* > 0.05) indicates no significant difference; Tukey’s HSD test. Different letters above bars indicate significant differences based on Tukey’s HSD test (*p* < 0.05).

With respect to ovarian development, relative to the control group, adults from the F_0_ generation in insecticide-treated groups exhibited significant reductions in vitellarium length on days 5 (*F*_2, 177_ = 18.68, *p* < 0.001) and 7 (*F*_2, 177_ = 10.52, *p* < 0.001) (Figure 2a), whereas comparable differences were not evident when comparing the control and insecticide-treated F_1_ generations from days 1 to 9 (1, *F*_2, 177_ = 1.08, *p* = 0.34; 3, *F*_2, 177_ = 2.87, *p* = 0.06; 5, *F*_2, 177_ = 2.60, *p* = 0.08; 7, *F*_2, 177_ = 2.76, *p* = 0.07; 9, *F*_2, 177_ = 1.44, *p* = 0.24) (Figure 2b). Significant differences in lateral oviduct diameter were also observed between treatment and control groups in adults from the F_0_ generation on day 5 (*F*_2, 177_ = 97.83, *p* < 0.001) (Figure 2c), while no significant differences in lateral oviduct diameter were observed in the LCT- or EMB-treated groups from the F_1_ generation at any analyzed time point relative to the control group (1, *F*_2, 177_ = 2.70, *p* = 0.07; 3, *F*_2, 177_ = 2.50, *p* = 0.09; 5, *F*_2, 177_ = 2.78, *p* = 0.07; 7, *F*_2, 177_ = 2.84, *p* = 0.06; 9, *F*_2, 177_ = 1.42, *p* = 0.25) (Figure 2d).

In addition, qRT-PCR analysis results revealed extremely low *Vg* transcript levels in adults on days 1 and 3, followed by pronounced increases in these levels on days 5, 7, and 9. Relative to the control group, *Vg* expression levels did not differ significantly in insecticide-treated groups on days 1 (*F*_2, 6_ = 1.19, *p* = 0.37) or 3 (*F*_2, 6_ = 0.46, *p* = 0.65) in the F_0_ generation, although they were significantly lower on days 5 (*F*_2, 6_ = 15.09, *p* = 0.005), 7 (*F*_2, 6_ = 9.03, *p* = 0.016), and 9 (*F*_2, 6_ = 32.37, *p* = 0.001) in these treatment groups (Figure 2e). Similar results were also observed for the F_1_ generation. Exposure to both LCT and EMB was associated with significant reductions in *Vg* expression on days 5 (*F*_2, 6_ = 5.87, *p* = 0.04) and 9 (*F*_2, 6_ = 7.08, *p* = 0.03) relative to the control group (Figure 2f).

### 3.5. Sublethal Effects of LCT and EMB on the Body Weight and Morphology of R. pedestris in the F_0_ and F_1_ Generations

**Figure 3:**
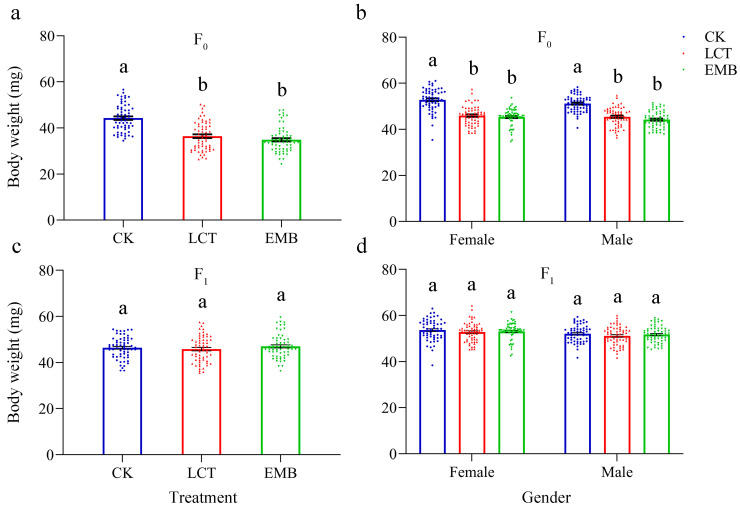
The sublethal effects of LCT and EMB on *R. pedestris* body weight. The body weight of (**a**) fifth instar nymphs from the F_0_ generation, (**b**) adults from the F_0_ generation, (**c**) fifth instar nymphs from the F_1_ generation, and (**d**) adults from the F_1_ generation. Three replicates (*n* = 20 individuals/replicate) were used for fifth instar nymphs, female, and male in each group. Blue circles, red regular triangles and green inverted triangles, respectively, represent values in the CK, LCT, and EMB groups. Bars with different letters indicate significant differences (*p* < 0.05) as determined via Tukey’s HSD test.

**Figure 4:**
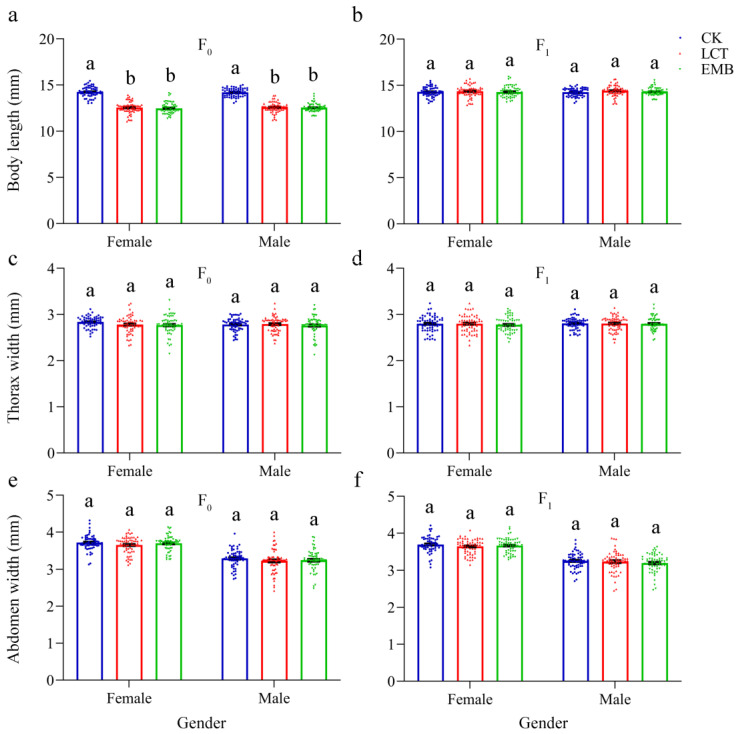
The sublethal effects of LCT and EMB on *R. pedestris* morphological parameters. The body length of adults in the F_0_ (**a**) and F_1_ (**b**) generations. The thorax width of adults in the F_0_ (**c**) and F_1_ (**d**) generations. The abdomen width of adults in the F_0_ (**e**) and F_1_ (**f**) generations. Three replicates (n = 20 individuals/replicate) were used for each treatment and control of each sex. Blue circles, red regular triangles, and green inverted triangles, respectively, represent values in the CK, LCT, and EMB groups. Bars with different letters indicate significant differences (*p* < 0.05) as determined via Tukey’s HSD test.

Relative to the control group (44.29 ± 0.76 mg), the body weight of fifth instar nymphs in the F_0_ generation in the insecticide-treated groups (LCT, 36.43 ± 0.81 mg; EMB, 34.87 ± 0.71 mg) were significantly reduced (*F*_2, 177_ = 44.02, *p* < 0.001) following the exposure of third instar nymphs to LC_30_ concentrations of LCT and EMB (Figure 3a), and these body weight reductions were observed irrespective of sex (male: *F*_2, 177_ = 60.37, *p* < 0.001; female: *F*_2, 177_ = 57.11, *p* < 0.001) (Figure 3b). Both males (*F*_2, 177_ = 224.27, *p* < 0.001) and females (*F*_2, 177_ = 175.39, *p* < 0.001) in the LCT and EMB treatment groups also exhibited significant reductions in body length relative to the control group (Figure 4a), although no differences in thorax (male: *F*_2, 177_ = 0.54, *p* = 0.58; female: *F*_2, 177_ = 2.84, *p* = 0.06) or abdomen width (male: *F*_2, 177_ = 0.82, *P* = 0.44; female: *F*_2, 177_ = 1.40, *p* = 0.25) were noted when comparing treatment and control groups (Figure 4c,e). In the F_1_ generation, no significant differences in the body weight of fifth instar nymphs (*F*_2, 177_ = 0.80, *p* = 0.45), male (*F*_2, 177_ = 0.92, *p* = 0.40), or female (*F*_2, 177_ = 0.68, *p* = 0.51) were noted among groups (Figure 3c,d), nor were there any significant alterations in adult body length (male: *F*_2, 177_ = 1.56, *p* = 0.21; female: *F*_2, 177_ = 0.32, *p* = 0.72), thorax width (male, *F*_2, 177_ = 0.07, *p* = 0.93; female, *F*_2, 177_ = 0.37, *p* = 0.69), or abdomen width (male, *F*_2, 177_ = 0.83, *p* = 0.44; female, *F*_2, 177_ = 1.04, *p* = 0.35) when comparing the insecticide-treated and control groups (Figure 4b,d,f).

### 3.6. Sublethal Effects of LCT and EMB on R. pedestris Population Parameters in the F_1_ Generation

Next, a detailed examination of the transgenerational impact of LCT and EMB exposure on *R. pedestris* population parameters in the F_1_ generation was conducted. Relative to the control group, a significant reduction in the net reproductive rate (*R*_0_) was observed for both insecticide treatment groups in the F_1_ generation (LCT, *p* = 0.044; EMB, *p* = 0.041). Similarly, significant differences in the intrinsic rate of increase (*r*) (LCT, *p* = 0.008; EMB, *P* = 0.012) and finite rate of increase (*λ*) values (LCT, *p* = 0.008; EMB, *p* = 0.012) were observed when comparing the treatment and control groups, although the mean generation time (*T*) (LCT, *p* = 0.051; EMB, *p* = 0.647) was comparable between groups in the F_1_ generation (Table 4).

**Figure 5:**
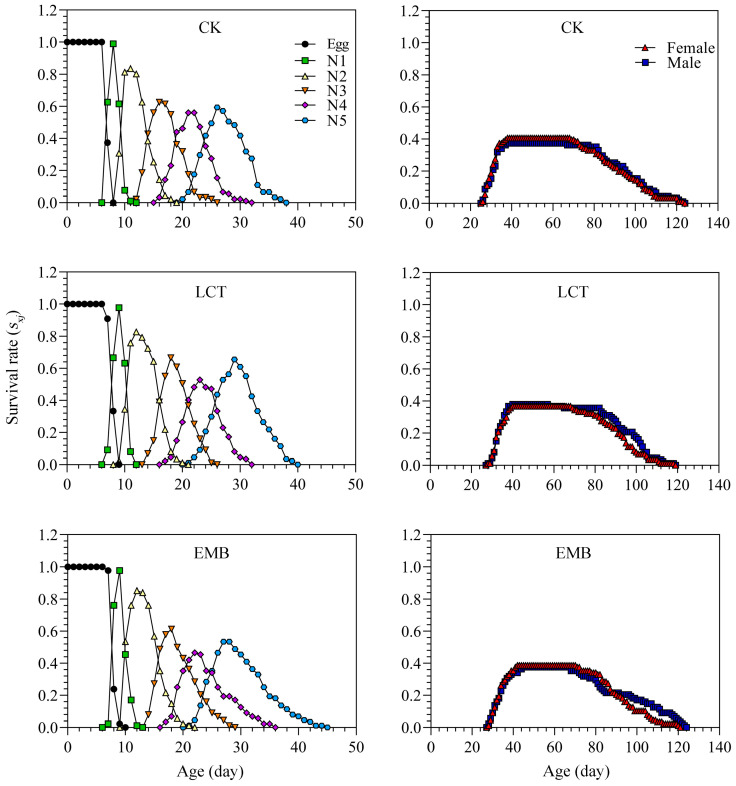
The age-stage specific survival rates (*s_xj_*) of *R. pedestris* in the F_1_ generation.

**Figure 6:**
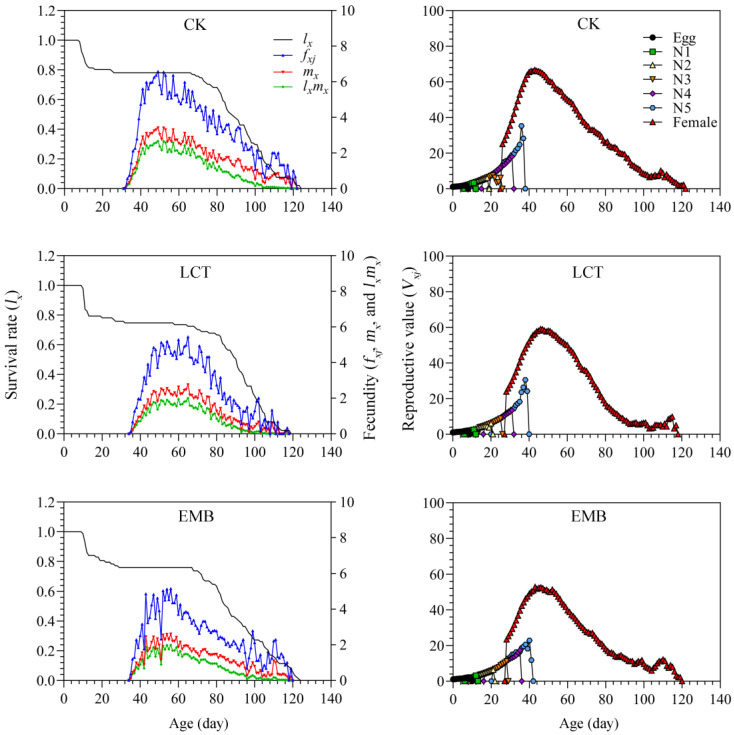
The age-specific survival rates (*l_x_*), age-stage specific fecundity (*f_xj_*), age-specific fecundity (*m_x_*), net maternity (*l_x_m_x_*), and reproductive value (*v_xj_*) of *R. pedestris* in the F_1_ generation.

**Figure 7:**
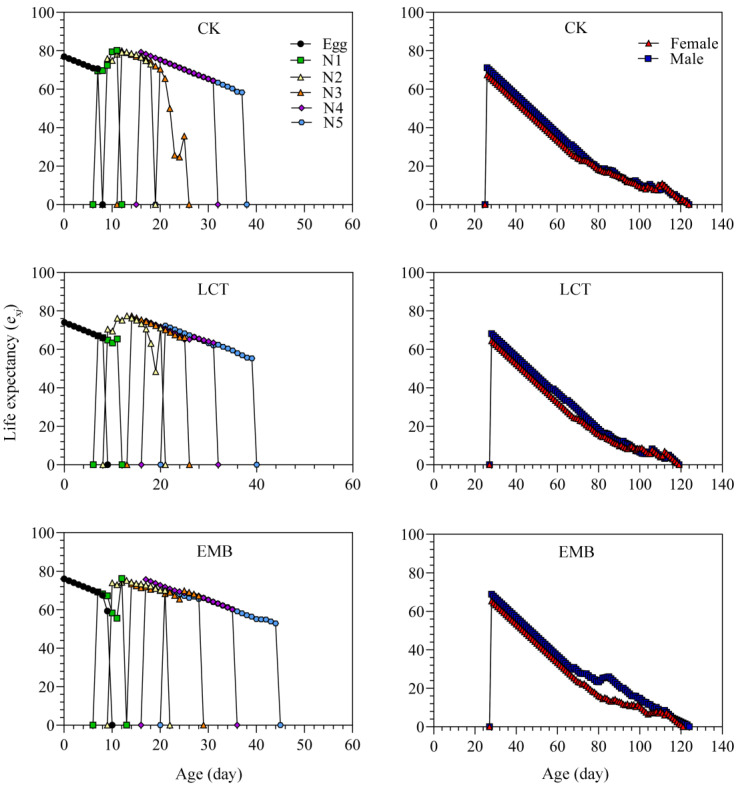
The age-stage specific life expectancy (*e_xj_*) of *R. pedestris* in the F_1_ generation.

Inter-individual variability with respect to developmental rates contributed to a pronounced overlap of stages when assessing the age-stage survival rate (*s_xj_*) values for *R. pedestris* in the control and insecticide-treated groups. Peak *s_xj_* values in the LCT-treated (third instar: 66.67%; female: 36.78%; male: 37.93%) and EMB-treated (third instar: 61.36%; female: 38.64%; male: 37.50%) groups were similar to those for the control group (third instar: 62.64%; female: 40.66%; male: 37.36%) (Figure 5). The age-specific survival rate (*l_x_*), which represents the odds of a newborn surviving to age *x* without taking stage into consideration, tended to decrease with age but did not differ significantly between the treatment and control groups. The highest age-stage-specific fecundity (*f_xj_*) values in the LCT- and EMB-treated groups were 5.44 and 5.15 eggs/female/day, respectively, and these values were below those for controls (6.57 eggs /female/day). The maximum age-specific fecundity (*m_x_*) in the LCT and EMB treatment groups was 2.76 and 2.61 eggs/individual/day, with this value was below that for controls (3.42 eggs/individual/day). The maximum age-specific maternity (*l_x_m_x_*) value for controls was also higher than that for the LCT and EMB treatment groups. Age-stage reproductive value (*v_xj_*) curves began to rise rapidly when females began laying eggs, peaking at a higher value for control females (66.75 eggs/female/day) as compared to those in the LCT (59.15 eggs/female/day) and EMB (52.71 eggs/female/day) groups (Figure 6). The maximal age-stage life expectancy (*e_xj_*) values from the egg stage to adulthood in the LCT (female, 64.44 days; male, 68.24 days) and EMB (female, 65.41 days; male, 68.97 days) groups were decreased relative to those of the control group (female, 67.49 days; male, 71.21 days) (Figure 7).

## 4. Discussion

Insecticides remain an essential component of IPM strategies as they can rapidly and effectively mitigate the harm associated with a broad range of pest species [34,35]. In agro-ecosystems, however, these pesticides are subject to asymmetrical application and degradation such that insects are frequently exposed to sublethal concentrations of these chemicals [36]. At sublethal concentrations, these compounds can still have profound effects on the biology, physiology, and biochemistry of exposed pests, thereby modulating overall population dynamics [7,12]. Efforts to assess the lethal and sublethal effects of different insecticides can inform efforts to rationally apply these compounds, facilitating the improvement of IPM strategies so as to minimize the risk of environmental harm [7]. Here, both LCT and EMB were found to be highly toxic to *R. pedestris* individuals, exhibiting concentration-dependent repellant effects. Sublethal LC_30_ concentrations of both LCT and EMB had negative impacts on the developmental duration, body weight, body length, and reproductive traits of *R. pedestris* in the F_0_ generation. Strikingly, these sublethal effects were also partially transmitted to the F_1_ generation.

Both LCT and EMB have been found to exhibit pronounced toxic effects on a range of insect species including *C. pomonella* [7], *A. lucorum* [18], and *M. separata* [19]. In this study, the LC_50_ value of LCT following the exposure of third instar *R. pedestris* nymphs for 72 h was 2.68 mg/L, consistent with its pronounced toxicity. The cypermethrin has similarly been shown to exhibit a high degree of toxicity against this pest species [37]. The LC_50_ value of EMB (7.68 mg/L) was 1.87-fold higher than that of LCT, potentially owing to the fact that these insecticides exhibit different modes of action. While the lethality of these compounds is important, there is also a pressing need to better understand the behavioral responses of pests to insecticides [38]. Here, third instar *R. pedestris* nymphs exhibited clear avoidance of LCT and EMB, in line with data from Romero et al. [39], who found that both individuals and groups of *Cimex lectularius* (L., 1758) (Hemiptera: Cimicidae) avoided dwelling on insecticide-treated filter paper. Maharjan and Jung [40] similarly found that *R. pedestris* adults avoided nine different insecticides, which included γ-cyhalothrin and cyfluthrin. These data highlight the repellant efficacy of these different compounds under laboratory conditions, providing insight that may guide their application in the context of IPM strategies directed against *R. pedestris*.

In addition to their direct lethal and repellant effects, insecticides have been shown to have a marked impact on insect development even at sublethal concentrations [7,13]. Here, the exposure of *R. pedestris* third instar nymphs to LCT and EMB resulted in the significant prolongation of the developmental duration in the nymph stage, while adult longevity was shortened notably as compared to controls. Pyrethroids or avermectins have been reported to exhibit similar sublethal effects in various mite and pest species. Moustafa et al. [23], for example, found that EMB exposure resulted in the significant prolongation of *M. brassicae* larval development, while Zhang et al. [41] determined that exposing *Rhopalosiphum padi* (L., 1758) (Hemiptera: Aphididae) to beta-cypermethrin at LC_20_ and LC_25_ doses respectively prolonged first instar nymph development and reduced the longevity of adults. Khan et al. [42] also observed increases in the duration of larval development and a corresponding reduction in male longevity following the exposure of *Panonychus citri* (McGregor, 1916) (Acari: Tetranychidae) to EMB at an LC_30_ concentration. Significant reductions in the body weight of both fifth instar nymphs and adults were also evident in the insecticide treatment groups relative to controls, in line with findings reported previously for insect species including *C. pomonella* [7], *S. littoralis* [24], and *Paederus fuscipes* Curtis, 1840, (Coleoptera: Staphylinidae) [43]. Two different factors may explain these results. First, insecticide intake can cause injury to the midgut, resulting in digestive dysfunction and metabolic disorder [21], thereby reducing food consumption and nutrient intake. Secondly, insecticide-exposed nymphs devote more energy to detoxification and various other defense mechanisms such as antioxidant defenses at the expense of growth and development [44,45]. Further research will be essential to clarify the intrinsic mechanisms that underlie LCT- and EMB-induced developmental inhibition.

Reproduction-associated parameters are also key metrics for use when assessing the sublethal effects of insecticides when used to treat arthropods [7,12,24]. Here, sublethal LCT and EMB concentrations resulted in APOP prolongation and reductions in oviposition days and fecundity in adult *R. pedestris* relative to the control group. The inhibitory effects of insecticides have similarly been observed in prior reports. Pre-oviposition period delays and decreases in the fecundity of adult *Nilaparvata lugens* (Stål, 1854) (Hemiptera: Delphacidae) were similarly observed following the exposure of second instar larvae to an LC_30_ concentration of EMB [43]. EMB LC_5_ and LC_15_ concentrations also resulted in significant APOP prolongation and a reduction in both *S. littoralis* oviposition days and fecundity [24]. LCT treatment also reduced the mean fecundity and oviposition period of *C. pomonella* at the LC_30_ concentration [7]. These results align well with the effects of sublethal LCT and EMB concentrations on *R. pedestris* population growth observed herein. The vitellin (Vn) precursor vitellogenin (Vg) plays a central role in the regulation of reproductive activity in insects such that measuring *Vg* expression can offer insight into the dynamic mechanisms governing female fecundity [25,33]. Here, the exposure of third instar nymphs to LC_30_ concentrations of LCT and EMB resulted in the suppression of *Vg* mRNA expression in adult *R. pedestris*. Decreased *Vg* levels, together with the observed reduction in fecundity in the insecticide treatment groups, may highlight the ability of these insecticides to modulate *R. pedestris* reproduction in part via regulating *Vg* expression. This result is consistent with the observed decreases in vitellarium length and lateral oviduct diameter values in the insecticide treatment groups as compared to the control group. Comparable findings have also been reported in *C. pomonella* [7] and *C. sinensis* [25]. These results are not universal, however, with significant increases in the fecundity and *Vg* expression levels of female *M. separata* having been observed following exposure to LCT at an LC_50_ concentration [19]. LC_25_ concentrations of triazophos were also associated with significant increases in fecundity and *Vg* levels in *S. furcifera* [26]. These discrepant results may be attributable to a range of factors, such as species, insecticide mode of action, and utilized insecticide concentrations [36].

To assess whether the sublethal effects of LCT and EMB can persist into the next generation when used to treat parental *R. pedestris,* developmental and reproductive parameters were also evaluated for the F_1_ generation. Following the exposure of the F_0_ generation to LC_30_ concentrations of LCT and EMB, a significant prolongation of development in the oviposition days and fecundity of adults relative to controls was observed. This is consistent with prior reports for afidopyropen-treated *A. gossypii* [11], spinetoram-treated *P. xylostella* [12], and pyriproxyfen-treated *Musca domestica* (L., 1758) (Diptera: Muscidae) [46]. These transgenerational effects are likely the result of epigenetic processes rather than mutations [2]. Exposing insects to sublethal insecticide concentrations has been shown to modulate gene expression through epigenetic processes including histone modifications and DNA methylation [2], with these modifications being transmissible to offspring [47], thus resulting in physiological changes via the process of transgenerational epigenetic inheritance [48]. The fecundity of the insecticide-treated group was greater for the F_1_ generation relative to the F_0_ generation, potentially suggesting that these transgenerational effects may weaken across generations such that they may ultimately disappear. However, further research will be needed to confirm this possibility.

Demographic parameters derived from two-sex life table analyses can offer comprehensive insights into insect population dynamics [49]. Here, exposure to LCT and EMB at the LC_30_ concentration level resulted in significant decreases in the net reproduction (*R*_0_), intrinsic rate of increase (*r*), and finite rate of increase (*λ*) values of members of the F_1_ generation in the insecticide-treated group relative to the control F_1_ population. In line with these data, Zhang et al. [41] previously observed a pronounced reduction in these demographic parameters in the F_1_ generation of *R. padi* after exposure of the parental generation of aphids to beta-cypermethrin at an LC_10_ concentration level. Moreover, Ghramh et al. [50] reported significant decreases in demographic parameters of the F_1_ generation of *M. domestica* following parental exposure to LC_10_ and LC_30_ concentrations of LCT relative to the control group. Members of the F_1_ generation in the insecticide-treated groups also exhibited significantly reduced *f_xj_*, *m_x_*, *l_x_m_x_*, *e_xj_*, and *v_xj_* values relative to controls. Overall, these data indicate that sublethal LCT and EMB concentrations can suppress *R. pedestris* population growth even in the offspring generation.

## 5. Conclusions

The present results revealed that both LCT and EMB caused significant toxicity and exhibited concentration-dependent repellent effects. Exposing third instar nymphs to these two insecticides resulted in significant increases in the duration of nymph development together with significant reductions in longevity, body weight, oviposition days, fecundity, and *Vg* expression for adult *R. pedestris* in the F_0_ generation. Exposure to LC_30_ concentrations of LCT and EMB also resulted in transgenerational suppressive effects in *R. pedestris* offspring. These data suggest that LCT and EMB can effectively inhibit *R. pedestris* population growth such that they may be effective tools for the control of *R. pedestris* infestations. As field conditions are more complex and variable than laboratory settings, however, additional research will be essential to validate and expand upon these results. Additional transcriptomic and metabolomics analyses will also be vital to clarify the mechanistic basis for the sublethal effects of LCT and EMB on *R. pedestris*.

## Figures and Tables

**Table 1:** The toxic effects of lambda-cyhalothrin (LCT) and emamectin benzoate (EMB) on the *R. pedestris* 3rd instar nymphs.

Insecticide	Slope ± SE ^a^	LC_30_ mg L^−1^ (95% CL) ^b^	LC_50_ mg L^−1^ (95% CL) ^b^	LC_70_ mg L^−1^ (95% CL) ^b^	LC_90_ mg L^−1^ (95% CL) ^b^	*χ* ^2^	*p*
lambda-cyhalothrin	1.227 ± 0.136	1.002 (0.649–1.369)	2.680 (2.058–3.404)	7.166 (5.523–9.998)	29.655 (19.061–57.087)	3.335	0.503
emamectin benzoate	1.309 ± 0.138	3.053 (2.107–4.026)	7.681 (6.057–9.620)	19.323 (15.051–26.588)	73.207 (48.307–133.801)	5.199	0.267

^a^ Standard error, ^b^ 95% confidence intervals.

**Table 2:** Sublethal effects of LCT and EMB on the development duration, longevity, and reproductive parameters of the F_0_ generation of *R. pedestris*.

Stages or Parameters	Control (CK) (*n*)	Lambda-Cyhalothrin (LCT) (*n*)	Emamectin Benzoate (EMB) (*n*)
Third instar (d)	4.96 ± 0.10 b (189)	6.63 ± 0.10 a (160)	6.85 ± 0.09 a (151)
Fourth instar (d)	5.03 ± 0.09 c (183)	7.30 ± 0.10 a (154)	5.68 ± 0.11 b (145)
Fifth instar (d)	6.67 ± 0.08 b (183)	7.83 ± 0.09 a (154)	7.57 ± 0.10 a (145)
Female longevity (d)	65.43 ± 1.65 a (60)	43.88 ± 1.37 b (60)	41.22 ± 1.38 b (60)
Male longevity (d)	73.25 ± 1.73 a (60)	52.32 ± 1.56 b (60)	49.73 ± 1.63 b (60)
APOP ^a^ (d)	6.37 ± 0.16 c (60)	8.47 ± 0.21 b (60)	8.67 ± 0.19 a (60)
Oviposition days (d)	50.38 ± 1.66 a (60)	30.20 ± 1.30 b (60)	24.00 ± 1.31 c (60)
Fecundity (eggs/female)	262.48 ± 8.57 a (60)	163.92 ± 7.67 b (60)	146.12 ± 7.46 b (60)

Development duration refers to the nymph developmental time (d). Reproductive parameters refers to APOP, oviposition day, and fecundity. Data are means ± SE. Different letters in a given row indicate significant differences based on Tukey’s HSD test (*p* < 0.05). ^a^ Adult pre-oviposition period.

**Table 3:** Sublethal effects of LCT and EMB on the development duration, longevity, and reproductive parameters of the F_1_ generation of *R. pedestris*.

Stages or Parameters	Control (CK) (*n*)	Lambda-Cyhalothrin (LCT) (*n*)	Emamectin Benzoate (EMB) (*n*)
Egg period (d)	7.37 ± 0.05 b (91)	8.24 ± 0.07 a (87)	8.23 ± 0.06 a (88)
First instar (d)	2.36 ± 0.06 a (81)	2.40 ± 0.06 a (75)	2.36 ± 0.07 a (78)
Second instar (d)	4.99 ± 0.16 b (74)	5.96 ± 0.15 a (68)	5.97 ± 0.17 a (74)
Third instar (d)	4.87 ± 0.12 a (71)	4.90 ± 0.18 a (68)	5.03 ± 0.19 a (69)
Fourth instar (d)	4.94 ± 0.12 a (71)	4.86 ± 0.11 a (66)	5.01 ± 0.13 a (69)
Fifth instar (d)	6.32 ± 0.13 b (71)	7.37 ± 0.15 a (65)	7.15 ± 0.14 a (67)
Preadult (d)	30.86 ± 0.36 b (71)	33.68 ± 0.36 a (65)	33.85 ± 0.50 a (67)
Female longevity (d)	62.65 ± 2.30 a (37)	58.78 ± 2.04 a (32)	59.71 ± 2.14 a (34)
Male longevity (d)	66.32 ± 2.22 a (34)	62.55 ± 2.27 a (33)	62.97 ± 3.15 a (33)
Total longevity (d)	95.27 ± 1.69 a (71)	94.37 ± 1.55 a (65)	95.16 ± 1.93 a (67)
APOP ^a^	6.57 ± 0.16 a (37)	7.19 ± 0.38 a (32)	6.85 ± 0.23 a (34)
TPOP ^b^	37.41 ± 0.49 b (37)	40.84 ± 0.70 a (32)	40.56 ± 0.71 a (34)
Oviposition days (d)	49.08 ± 2.04 a (37)	42.09 ± 1.41 b (32)	43.32 ± 1.77 b (34)
Fecundity (eggs/female)	253.43 ± 11.38 a (37)	191.56 ± 8.50 b (32)	176.97 ± 8.94 b (34)

Development duration refers to the egg, nymph developmental time (d). Reproductive parameters refers to APOP, TPOP, oviposition day, and fecundity. Data are means ± SE. Different letters in a given row indicate significant differences based on paired bootstrap tests (*p* < 0.05). ^a^ Adult pre-oviposition period. ^b^ Total preoviposition period.

**Table 4:** Sublethal effects of LCT and EMB on the population parameters of the *R. pedestris* F_1_ generation.

Parameters	Control (CK)	Lambda-Cyhalothrin	Emamectin Benzoate
Net reproductive rate (*R*_0_)	103.044 ± 13.847 a	70.460 ± 10.368 b	68.375 ± 9.819 b
Intrinsic rate of increase (d^−1^) (*r*)	0.086 ±0.029 a	0.075 ± 0.030 b	0.075 ± 0.031 b
Finite rate of increase (d^−1^) (*λ*)	1.090 ± 0.032 a	1.077 ± 0.032 b	1.078 ± 0.033 b
Mean generation time (d) (*T*)	54.023 ± 0.765 a	57.035 ± 0.897 a	56.355 ± 1.162 a

Data are means ± SE. Different letters in a given row indicate significant differences based on paired bootstrap tests (*p* < 0.05).

## Data Availability

The data presented in this study are available on request from the corresponding author. The data are not publicly available due to confidentiality.

## Supplementary Materials

The following supporting information can be downloaded at: https://www.mdpi.com/article/10.3390/toxics11120971/s1, Figure S1: The experimental methodology flow.
