# Supplementary figures and images for: The Lethal and Sublethal Effects of Lambda-Cyhalothrin and Emamectin Benzoate on the Soybean Pest Riptortus pedestris (Fabricius)
Source: Toxics. 2023 Nov 30;11(12):971. doi: 10.3390/toxics11120971 (PMC10747274; doi:10.3390/toxics11120971)

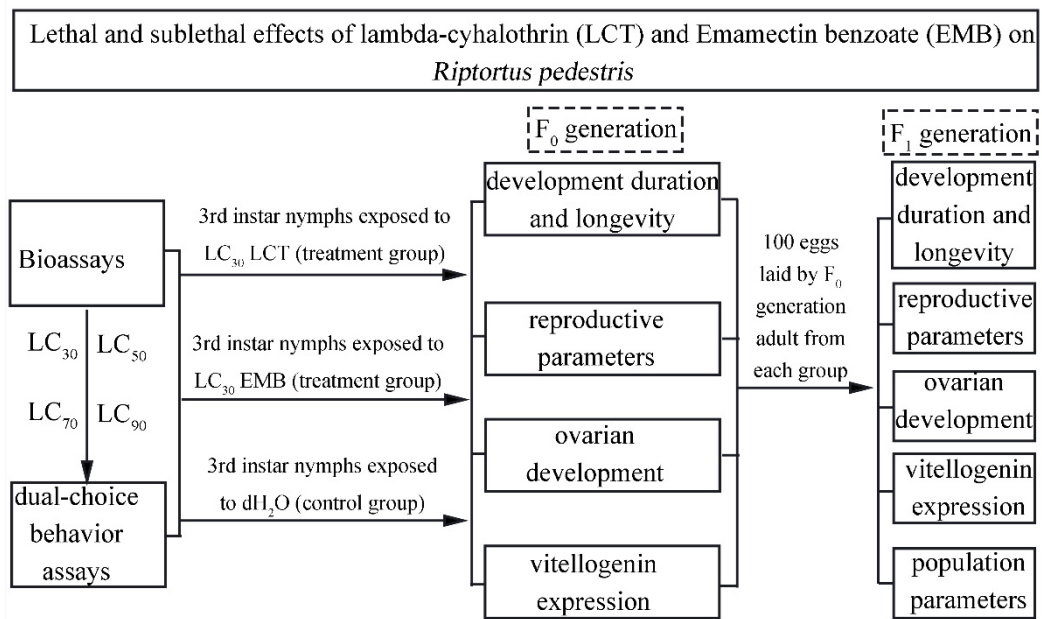

**Figure S1.** The experimental methodology flow.

Supplement: Supplementary file 1 [file toxics-11-00971-s001.zip › toxics-2716878-supplementary.pdf]
